# Testing Geology with Biology: Plate Tectonics and the Diversification of Microhylid Frogs in the Papuan Region

**DOI:** 10.1093/iob/obad028

**Published:** 2023-07-29

**Authors:** Ethan C Hill, Diana F Gao, Dan A Polhemus, Claire J Fraser, Bulisa Iova, Allen Allison, Marguerite A Butler

**Affiliations:** School of Life Sciences, University of Hawai‘i, 2538 McCarthy Mall, Honolulu, 96822, HI, USA; School of Life Sciences, University of Hawai‘i, 2538 McCarthy Mall, Honolulu, 96822, HI, USA; Department of Biology, University of San Francisco, 2130 Fulton St, Harney Science Center, San Francisco, 94117, CA, USA; Natural Science, Bernice Pauahi Bishop Museum, Street, 96817, HI, USA; School of Life Sciences, University of Hawai‘i, 2538 McCarthy Mall, Honolulu, 96822, HI, USA; National Museum and Art Gallery, Boroko, National Capital District, PNG; Natural Science, Bernice Pauahi Bishop Museum, Street, 96817, HI, USA; School of Life Sciences, University of Hawai‘i, 2538 McCarthy Mall, Honolulu, 96822, HI, USA

## Abstract

Studies of the Papuan region have provided fundamental insights into the evolutionary processes generating its exceptional biodiversity, but the influence of geological processes merits further study. Lying at the junction of five tectonic plates, this region has experienced a turbulent geological history that has not only produced towering mountains allowing elevational specialization and island archipelagos with varying degrees of isolation promoting vicariance, but also active margins where land masses have collided and been subsequently rifted apart creating a mosaic of intermixed terranes with vastly different geological histories. Asterophryine frogs are a hyperdiverse clade representing half the world’s microhylid diversity (over 360 species) centered on New Guinea and its satellite islands. We show that vicariance facilitated by geological history explains this far and wide distribution of a clade that should have poor dispersal abilities. We recovered a mainland tectonic unit, the East Papua Composite Terrane (EPCT), as the center of origin for Asterophryinae and no fewer than 71 instances of what appear to be long-distance dispersal events, 29 of which are between mainland regions, with 42 from the mainland to the islands, some presently as far as 200 km away from source populations over open ocean. Furthermore, we find strong support for a “Slow and Steady” hypothesis for the formation of the northern margin of New Guinea by many separate accretion events during the Miocene, over other major geological alternatives, consistent with the 20 M year age of the clade and arrival via the EPCT. In addition, the historical biogeography of our frogs strongly supports an affiliation of the Louisiade Archipelago and Woodlark Island with the Owen Stanley Range on the EPCT, and the recent proximity of the large New Britain Island. Our results show that Asterophryinae did not have to repeatedly and independently disperse across large ocean barriers to the offshore islands, against the predictions of island biogeography theory, but that the current distribution can be explained through vicariance and short-distance oceanic dispersal as historical land connections disappeared and islands slowly became separated from each other. We show that islands have a life history, changing in distance from other land masses, with consequent opportunities for dispersal, isolation, and cladogenesis of their biotas. More broadly, we can begin to see how the geological history of the Papuan region can result in the rapid accumulation and staggering number of extant species.

*“The distributions of organisms not adapted for long-distance dispersal are good evidence of past land connections.”*

— Biogeographical principles advocated by Sir Alfred Russel Wallace Source: Brown and Lomolino (1998)

Wallace (1876, 1880) proposed that to understand the fascinating distributional patterns in the South Pacific requires a consideration of both natural selection and earth history (Hazzi et al. 2018; Pellissier et al. 2018). The island of New Guinea hosts staggering biodiversity, containing among the highest degrees of endemism of any place on the earth (Allison, 2009; Brooks et al. 2006; Gressitt 1982) and provides a case in point, lying in an intersection zone between Asian and Australian faunas that coincides with tectonic provinces between the two areas. Wallace’s second proposal, a changing geology may shape the distribution and diversity of life, is supported by correlation between geological events and areas of high species richness. The rise of the central high mountains is associated with vicariance in ants (Li and Li 2018) and freshwater turtles (Georges et al. 2014), and signatures of presumed island fragmentation and accretion sequences are reflected in patterns of endemism in island insects (Boer and Duffels 1996; Polhemus 1996; Polhemus and Polhemus 1998). Recent advances in phylogenetic methods incorporating timing informed by new geological data for New Guinea (reviewed in: Baldwin et al. 2012) have inspired more explicit spatio-temporal hypotheses of distribution and diversity (e.g., Jønsson et al. 2011; Georges et al. 2014; Toussaint et al. 2014). Toussaint et al. (2014) found support for recent orogeny driving the diversification of montane arthropods, supporting the geological model of Hall (1998, 2002), which posits that collision between the Pacific and Australian plates resulted in uplift of the central cordillera and the rest of mainland New Guinea. We call this model “recent emergence” as it predicts that as late as (∼5Ma), most of New Guinea was under water with only the central high mountains above sea level and available for colonization by terrestrial taxa. While this model explains diversity patterns of young taxa centered on the central cordillera, there are both additional major biogeographical patterns as well as alternative hypotheses of geological history, which make different spatio-temporal predictions. More generally, biological data has the—thus far mostly untapped—potential to inform competing geological hypotheses, but what is required is consideration of alternative geological hypotheses that are tested with clades that are older and more widely distributed across New Guinea (but see: Cozzarolo et al. 2019).

New Guinea lies in one of the most tectonically active regions of the world, and while its geological history is complex, it is generally accepted that the formation of the island involves the collision of multiple plates and accretion of multiple island arcs (reviewed in: Baldwin et al. 2012). Starting along the northern edge of the Australian Craton, the island formed over the Cenozoic with the additions of the East Papua Composite Terrane, the Fold Belt, the Accreted Terranes, and the Vogelkop Peninsula (Dow 1977; Pigram and Davies 1987; Pigram and Symond 1991; Crowhurst et al. 1996; Davies et al. 1996, 1997; Hall 2002; Quarles van Ufford and Cloos 2005; Baldwin et al. 2012; Webb et al. 2014; Holm et al. 2019). These processes were initiated by the collision of the northward-moving Australian Plate with the west-northwest-moving Pacific Plate, with the additional interactions of the smaller Philippine, Caroline, and Solomon Sea plates (Kroenke et al. 1984; Kroenke 1996; Hall 1998; Klootwijk et al. 2003), and various other microplates that were shattered off along their edges during convergence. This resulted in sequential accretion of island arcs and subduction or obduction of oceanic and continental crust (Polhemus 2007; Baldwin et al. 2012), to form an island of large size and complex topography, as well as a specific temporal ordering of land connections. The last major addition occurred within the last several million years, involving the rotation and subduction of the South Bismarck Plate at the northern margin to form the most recent component of the Accreted Terranes, adding the Adelbert-Finnisterre terrane containing the Huon Peninsula, and bringing the New Britain Island arc into the closest proximity to the mainland that it has ever been (Baldwin et al. 2012).

In addition to the “recent emergence” hypothesis, two additional geological hypotheses have particular relevance for biotic diversification and differ with regard to the timing and assembly of the northern coastal terranes. The “Mobile Belt” hypothesis posits that the Accreted Terranes were assembled offshore into a single unit during the Late Oligocene (25–23 Ma), and subsequently docked onto the growing mainland in the mid-Miocene (15–11 Ma; Dow 1972, 1977; Davies 2012) giving rapid rise to the central high mountains in the Fold Belt (Quarles van Ufford and Cloos 2005; Webb et al. 2014; Holm et al. 2019). Alternatively, several authors hypothesize that accretion along the northern coast has been a “slow and steady” process extending from the Late Cretaceous to the Pleistocene (Pigram and Davies 1987; Davies et al. 1996; Quarles van Ufford and Cloos 2005). Under this scenario, the ECPT is a composite of terranes, which accreted onto a displaced sliver of the Australian Craton beginning with an arc collision in the Paleocene/Eocene, and then suturing to the main body of New Guinea in the Late Oligocene to Middle Miocene (30–25 Ma, Davies et al. 1997), while the addition of the Accreted Terranes in the northern part of mainland New Guinea began in the Paleocene in the west (68 Ma) and extended until the Pliocene (5–2 Ma) in the east, the most recent ongoing addition being portions of the New Britain Island arc. The orogeny of the central high mountains is proposed to have occurred in the mid-Miocene (15–11 Ma), together with docking of the Vogelkop Peninsula in the west (Pigram and Davies 1987; Polhemus and Polhemus 1998; Quarles van Ufford and Cloos 2005; Davies 2012), although Holm et al. (2019) hypothesize that Vogelkop sutured onto the western edge of New Guinea more recently, in the Late Miocene (7–3 Ma). Each of these geological models provides very different spatio-temporal implications for the evolution of biodiversity.

Frogs of the subfamily Asterophryinae are a particularly appropriate group to study the role of geologic history on distribution and diversification. Nearly 700 species of microhylid frogs are distributed worldwide across the Americas, Africa, Asia, and Australia (AmphibiaWeb 2020), but half of them—over 360 recognized species—comprise its largest subfamily, Asterophryinae, which are centered in the Papuan region (New Guinea and its satellite islands, and the Bismarck and Louisiade Archipelagos), and extend into Malaysia, the Philippines, the northeastern coast of Australia, and have recently been described from Thailand to Vietnam (Poyarkov et al. 2018; Suwannapoom et al. 2018). Recent molecular studies indicate they arose during the Miocene, within the past ∼20 MY (Feng et al. 2017; Hill et al. 2022), coincident with major geological development of New Guinea. High generic diversity (17 recognized genera) and endemism of Asterophryinae relative to the other four native anuran families in New Guinea suggest they were probably the first frog lineage to colonize the region (Savage 1973; Frost et al. 2006; Van Bocxlaer et al. 2006; van der Meijden et al. 2007; Köhler and Günther 2008; Rivera et al. 2017; Tu et al. 2018; Hill et al. 2022). Anurans are poor oceanic dispersers, yet a substantial number of species occurring on the offshore islands have deeply divergent sister taxon relationships with lineages on the New Guinea mainland (Hill et al. 2022), suggesting either long-distance overwater dispersal or past geologic connection between land masses.

In this study, we reconstruct the biogeographic history of Asterophryinae using a densely sampled phylogeny coupled with sophisticated evolutionary analyses to test geological hypotheses for diversification. We construct alternative geological hypotheses for (i) slow and steady accumulation of the Accreted and East Papua Composite Terranes, (ii) offshore Mobile Belt formation, (iii) recent emergence of New Guinea, and (iv) development of the offshore islands, which have received little geological study, and allow these hypotheses to compete for the best explanation of range evolution. We infer ancestral ranges and the minimum number of dispersal events to explain the biogeographic distribution of the clade.

## Materials and methods

### Taxonomic and geographic sampling

**Fig. 1:**
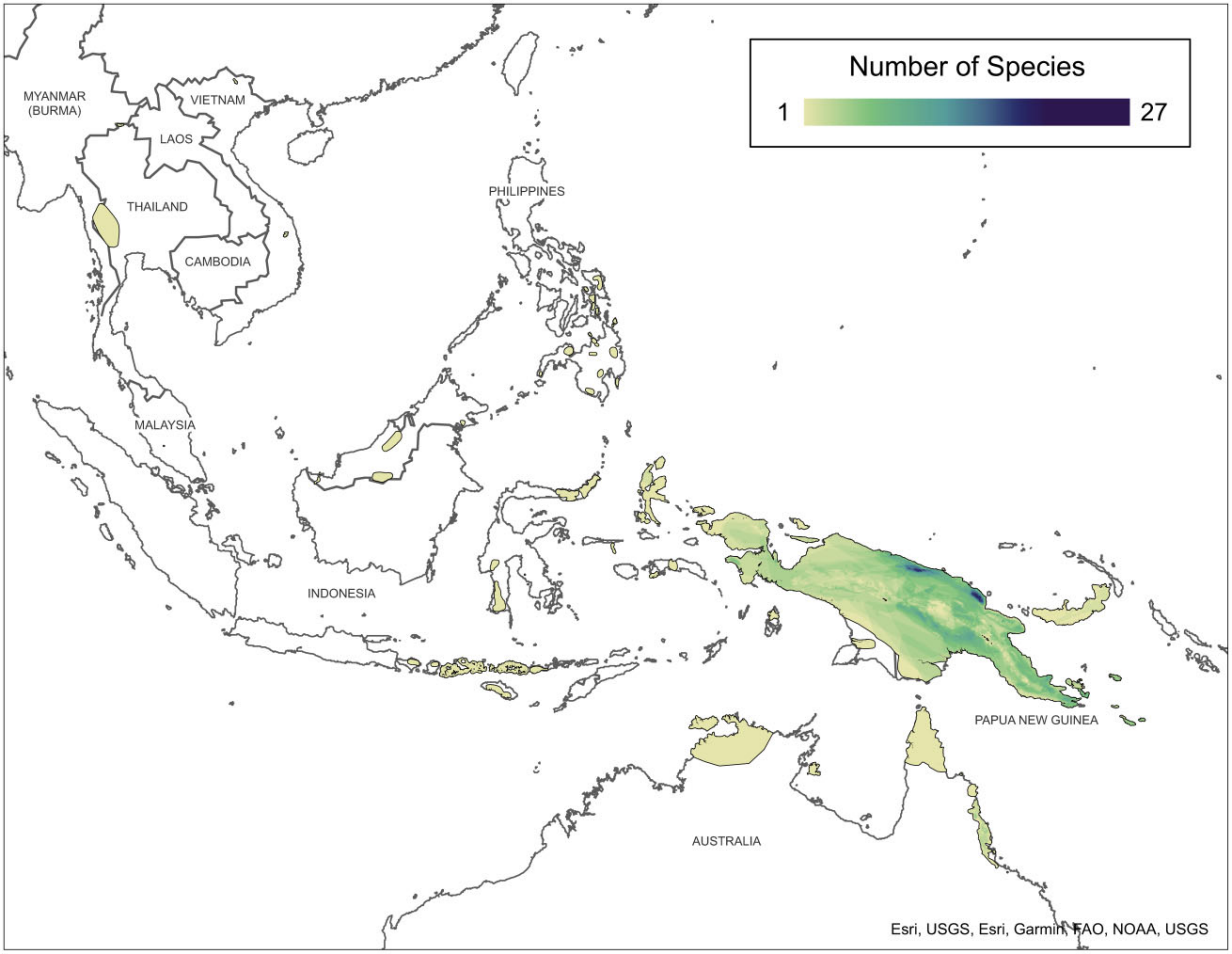
Distribution of species of Asterophryinae species based on shapefiles from the IUCN Red List (IUCN 2022).

The subfamily Asterophryinae is distributed across the Southwest Pacific (Fig. 1IUCN 2022). Over 90% of the diversity of Asterophryinae (327 species out of 363 named species) is endemic to the Papuan region including New Guinea Island and several of its satellite islands (the D’Entrecasteaux Islands, other small satellite islands, and the Louisiade Archipelago), with a few species occurring along the northern coast of Australia, in the Philippines, and in Indochina from Vietnam and Thailand southward into Malaysia. Asterophryines were almost certainly the first anuran lineage to colonize New Guinea as they are the dominant anuran fauna of New Guinea Island (especially concentrated along the northern coast of New Guinea) and several of its satellite islands, whereas they represent only minor portions of the anuran fauna in Asia and Australia.

**Fig. 2:**
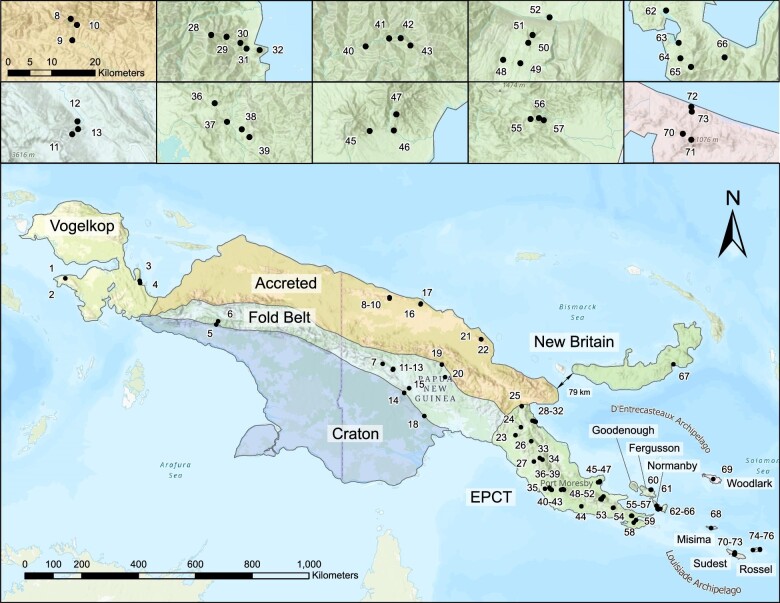
Five major geological regions of NG that illustrate accretion history. The island of New Guinea has a composite history with multiple geologic terranes alagamating to form the large island. The geologic terranes of New Guinea are labeled following Davies (2012): the East Papua Composite Terrane, the Accreted Terranes, the Fold Belt, the Australian Craton, and the Vogelkop Peninsula. Sampling sites across PNG and satellite islands are indicated by numbers, with insets showing detail for sites that are clustered. Site names, GPS coordinates, and metadata are provided in Table 1 of Hill et al. (2023).

For our biogeographical analysis, we used our previously published dataset (Hill et al. 2022, 2023), which contains 218 samples from over 80 sites across Papua New Guinea and its satellite islands (Fig. 2), spanning a majority of the geographic extent of Asterophryinae, and reasonably representing the known geographic diversity (Fig. 1). Importantly, this geographic sampling includes all five tectonic sectors of mainland New Guinea as well as the majority of taxa from several satellite islands where Asterophryinae diversity is high (Normanby and Fergusson of the D’Entrecasteux Islands, Misima, Rossel, and Sudest Islands of the Louisiade Archipelago, Woodlark Island, and New Britain Island), with two samples each from the Philippines and Sulawesi, Indonesia.

We used the time-calibrated Asterophryinae phylogeny of Hill et al. (2022, 2023), which contains 218 tips and is the largest and most comprehensive phylogenetic effort for this subfamily to date. Time-calibration was made using widely-agreed upon geological references, specifically the isolation of the islands that form the Louisiade Archipelago (6–4 Ma) and the opening of the Woodlark Basin (6–5 Ma, Hill et al. 1992; Davies et al. 1997; Baldwin et al. 2012; Wallace et al. 2014; Webb et al. 2014); see Hill et al. (2022) and Rivera et al. (2017) for further explanation. Although no fossil data are available for Asterophryinae, the age of the tree is corroborated by the independent analysis of Feng et al. (2017), who recovered a 20-M-year old age estimate for Asterophryinae using 20 fossil age constraints from older amphibian groups.

This taxonomic sampling includes 205 taxa, 122 named species and an additional 83 putative taxa, and importantly includes robust sampling across all known genera (16 monophyletic genera and two historically recognized genera whose monophyly remains unconfirmed). We note that while Asterophryinae taxonomy is much improved based on recent molecular studies (van der Meijden et al. 2007; Köhler and Günther 2008; de Sa et al. 2012; Peloso et al. 2016; Rivera et al. 2017; Tu et al. 2018; Hill et al. 2022, 2023), the taxonomy is incomplete and complicated by a large number of undescribed species (a rough estimate is approximately 40% of collections are undescribed), as well as what is sure to be extensive undiscovered diversity (many recent studies report cryptic diversity across many lineages).

Our overall sampling strategy was to include as many geographically and taxonomically representative samples as we could obtain from our field studies spanning several decades along with contributions from colleagues (see acknowledgements in: Hill et al. 2022, 2023). Furthermore, we note that New Guinea Island contains two countries, and fieldwork has been more extensive in Papua New Guinea due to the logistical and political difficulties of working in Indonesia. Therefore, the full extent of Asterophryinae diversity is not completely known. However, this dataset contains the largest geographic and taxonomic sampling of the known distribution of Asterophryinae of the Papuan Region.

The geographical sampling includes 13 georegions used to test hypotheses of historical phylogeography: all 5 recognized major tectonic units of mainland Papua New Guinea: the East Papua Composite Terrane (E), the Fold Belt (F), the Australian Craton (A), the Accreted Terranes (A), and the Vogelkop Peninsula (V); 7 offshore islands including New Britain Island (B), Misima Island (M), Sudest Island (S), Rossel Island (R), Normanby Island (Y), Fergusson Island (G), Woodlark Island (W); and Southeast Asia (N). A total of 17 sites are located on offshore islands with the remaining 63 occurring on the Papuan mainland. A summary of the number of species and sites per georegion are listed in Table 1, with metadata for all samples including GPS coordinates provided in Table 1 of Hill et al. (2023). We focused our geological exploration on alternative hypotheses of shared geohistory among these regions.

**Table 1:** The number of representative taxa for each georegion included in this analysis

				Area	Distance
	Georegion	Taxa	Sites	(km^2^)	(km)
	New Guinea	159		785,753	
V	Vogelkop Peninsula	4	3		
A	Accreted Terranes	32	7		
F	Fold Belt	19	8		
C	Australian Craton	3	3		
E	EPCT	101	37		
Y	Normanby Island	18	6	1,040	40
F	Fergusson Island	3	1	1,437	40
B	New Britain	2	2	36,520	79
W	Woodlark Island	6	1	874	253
M	Misima Island	4	1	202	202
S	Sudest Island	8	4	866	303
R	Rossel Island	8	3	262	372

Island area data from (Lyon 1991) http://islands.unep.ch/Tiarea.htm. Distances are oceanic distances from islands to the nearest point on the mainland.

### Modeling the evolution of geographic range

We modeled the evolution of geographic ranges of Asterophryinae using dispersal-extinction-cladogenesis models (DEC Ree et al. 2005; Ree and Smith 2008), using the Bayesian implementation provided by Matzke (2014). These biogeographic models are described in detail in Ree et al. (2005) and Ree and Smith (2008), but we briefly review the key components here to explain our hypothesis testing approach, which required representing differences in dispersal opportunity afforded by the alternate geological scenarios. We use “areas” to refer to distinct georegions observed in our dataset, and “range” as the set of areas occupied by a lineage; thus, areas are fixed but species ranges can evolve through time. DEC models describe an evolutionary process for biogeographic range evolution along a phylogeny (Ree et al. 2005; Ree and Smith 2008), and are closely related to stochastic models for discrete character evolution, but importantly differ in breaking up each state change into two possible stochastic events: dispersal to a new area, and extinction from an existing area. Discrete changes from one state to another are assumed to occur randomly with respect to time according to a Markov process with probability matrix **P**_*ij*_(*t*) from ancestor state *i* to descendant state *j*. In matrix form this equation is:


\begin{equation*}
\mathbf {P}(t) = e^{-\mathbf {Q}(t)}.
\end{equation*}

Where **Q** is the instantaneous rate matrix containing both the rates of dispersal *D_ij_* between ancestor range *i* to descendant range *j*, and rates of local extinction *E_i_*. For example, for three areas (1, 2, 3, along with the null area ∅ for the possibility of extinction), and with each lineage occupying a maximum of two areas, the possible ranges would be enumerated as *S* = {∅, 1, 2, 3, 12, 13, 23}, and **Q** would be parameterized as


\begin{equation*}
\mathbf {Q} = \!\!\!\!\!\!\!
\begin{array}{r}
\emptyset \\
\quad 1\\
\quad 2\\
\quad 3\\
\quad 12\\
\quad 13\\
\quad 23
\end{array}
\begin{array}{l}
\quad\emptyset\quad\ \ \ 1\quad\ \ \ \ 2\quad\ \ \ 3\quad\ \ \ 12\quad\ \ \ 13\quad\ \ \ \ 23 \\
\left[\begin{array}{cccccccc}
- & \quad 0 & \quad 0 & \quad 0 & \quad 0 & \quad 0 & \quad 0 \\
E_{1} & \quad - & \quad 0 & \quad 0 & \quad D_{12} & \quad D_{13} & \quad 0 \\
E_{2} & \quad 0 & \quad - & \quad 0 & \quad D_{21} & \quad 0 & \quad D_{23} \\
E_{3} & \quad 0 & \quad 0 & \quad - & \quad 0 & \quad D_{31} & \quad D_{32} \\
0 & \quad E_{2} & \quad E_{1} & \quad 0 & \quad - & \quad 0 & \quad 0 \\
0 & \quad E_{3} & \quad 0 & \quad E_{1} & \quad 0 & \quad - & \quad 0 \\
0 & \quad 0 & \quad E_{3} & \quad E_{2} & \quad 0 & \quad 0 & \quad -
\end{array}\right]\end{array}.
\end{equation*}

Thus, a frog lineage may expand its range by dispersal into a new area, contract its range when extinction occurs from part of its range, or may change areas if both dispersal to the new and extinction from the old areas occur jointly. These equations model state changes along branches, with nodes assumed to represent speciation events. As the phylogeny is assumed to be true (as with many phylogenetic comparative methods), all possible state combinations at internal nodes compatible with the observed ranges at the tips are integrated over in computing a likelihood for the model. The model outputs include maximum likelihood estimates for the dispersal and extinction rates and inferred ancestral ranges at the nodes. DEC models are flexible enough to allow a variety of evolutionary outcomes, including rapid spread as well as slow biogeographical evolution across a landscape, as one would expect for lineages that are poor dispersers such as frogs, as opposed to free movement from anywhere to anywhere as would be allowed for standard discrete character models.

We focus on testing alternative geological hypotheses that explain the dispersal history of our frogs, implemented by adding constraints that represent hypothetical dispersal barriers. We used the dispersal multipliers matrix in BioGeoBears (Matzke 2014), with “1”s indicating each pair of areas where dispersal is allowed, and “0”s for each pair of areas where dispersal is highly unlikely, as may occur between islands separated by open ocean (we note in practice, the zeros are instead “0.001”s, a small number for the software to return a model fit). The dispersal elements of the Q matrix are multiplied by these constraints, influencing which dispersal paths dominate in the explanation of the data.

**Fig. 3:**
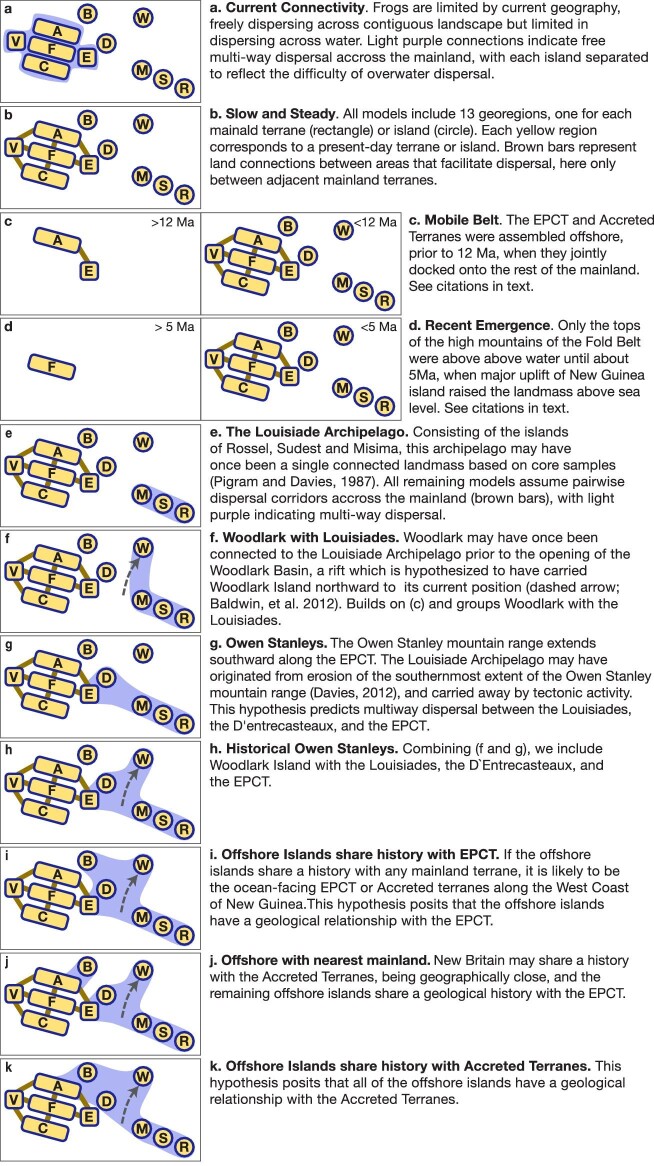
Visual representations of the alternative hypotheses. All models include 13 georegions indicated in yellow, one for each mainland terrane (rectangle) or island (circle). Note that the two islands of the D‘Entrecasteaux (Normanby and Fergusson) are represented by “D”, and the Southeast Asian georegion is not shown. Brown bars represent pairwise land connections between areas that facilitate dispersal, whereas areas grouped in light purple indicate multi-way connections. These alternatives are expressed as modifications to the dispersal multiplier matrix (see Methods). Hypotheses **b, c**, and **d** are the major alternatives of slow and steady, mobile belt, and recent emergence, respectively, versus the alternative “null” hypothesis of no geohistory **a**. Hypotheses **e–k** are further refinements of **b** incorporating the history of the offshore islands. Hypotheses **c** and **d** are time-stratified by areas and dispersal corridors available during the periods indicated.

We used dispersal multiplier matrices with either pairwise connections or multiway connections to represent our geological scenarios. In particular, New Guinea Island was formed by terranes docking or accreting onto existing terranes along particular margins, which was modeled with a pairwise connection matrix, with the EPCT connected to the Accreted Terranes and the Fold Belt with 1s (brown bars indicating connections in Fig. 3), but neither the Australian Craton nor the Vogelkop Peninsula (0s). We note that it is possible for EPCT lineages to reach the Vogelkop, but would require an additional step through the Accreted Terranes, for example, to create a connected path consistent with the difficulty of long-distance dispersal in this system. Alternatively, we have archipelagos, which may have been connected at one time, or a hypothesis of islands rifting off from a mainland origin, such as for the Louisiade Archipelago, which is hypothesized to have originated as a southeastern extension of the Owen Stanley Range of the EPCT prior to its current state as a set of separate oceanic islands. This hypothesis is represented by all members of the affected islands and the EPCT connected by “1”s to each other, forming a block of multi-way connections (light purple areas of connection in Fig. 3).

For example, hypothesis **a**, Naive Geohistory, in which only the mainland terrane connections are involved (potentially among V, C, F, A, E), is represented by the dispersal multiplier matrix below:


\begin{eqnarray*}
\mathbf {d_{a}} =
\begin{array}{c}
\\ V\\
C\\
F\\
A\\
E\\
D\\
W\\
M\\
S\\
R\\
B
\end{array}
\begin{array}{l}
\quad V \quad C \quad F\quad A\quad E\quad D\quad W\quad M\quad S\quad R\quad B \\
\left[\begin{array}{c@{}c@{}c@{}c@{}c@{}c@{}c@{}c@{}c@{}c@{}c}
1 &\quad 1 &\quad 1 &\quad 1 &\quad 0 &\quad 0 &\quad 0 &\quad 0 &\quad 0 &\quad 0 &\quad 0 \\
1 &\quad 1 &\quad 1 &\quad 0 &\quad 0 &\quad 0 &\quad 0 &\quad 0 &\quad 0 &\quad 0 &\quad 0 \\
1 &\quad 1 &\quad 1 &\quad 1 &\quad 1 &\quad 0 &\quad 0 &\quad 0 &\quad 0 &\quad 0 &\quad 0 \\
1 &\quad 0 &\quad 1 &\quad 1 &\quad 1 &\quad 0 &\quad 0 &\quad 0 &\quad 0 &\quad 0 &\quad 0 \\
0 &\quad 0 &\quad 1 &\quad 1 &\quad 1 &\quad 0 &\quad 0 &\quad 0 &\quad 0 &\quad 0 &\quad 0 \\
0 &\quad 0 &\quad 0 &\quad 0 &\quad 0 &\quad 1 &\quad 0 &\quad 0 &\quad 0 &\quad 0 &\quad 0 \\
0 &\quad 0 &\quad 0 &\quad 0 &\quad 0 &\quad 0 &\quad 1 &\quad 0 &\quad 0 &\quad 0 &\quad 0 \\
0 &\quad 0 &\quad 0 &\quad 0 &\quad 0 &\quad 0 &\quad 0 &\quad 1 &\quad 0 &\quad 0 &\quad 0 \\
0 &\quad 0 &\quad 0 &\quad 0 &\quad 0 &\quad 0 &\quad 0 &\quad 0 &\quad 1 &\quad 0 &\quad 0 \\
0 &\quad 0 &\quad 0 &\quad 0 &\quad 0 &\quad 0 &\quad 0 &\quad 0 &\quad 0 &\quad 1 &\quad 0 \\
0 &\quad 0 &\quad 0 &\quad 0 &\quad 0 &\quad 0 &\quad 0 &\quad 0 &\quad 0 &\quad 0 &\quad 1
\end{array}\right]
\end{array}.
\end{eqnarray*}

Whereas hypothesis **g** posits that a multi-way connection existed between all offshore islands and the EPCT, but to none of the remaining mainland terranes:


\begin{eqnarray*}
\mathbf {d_{g}} =
\begin{array}{c}
\\V\\C\\F\\ A\\ E\\ D\\ W\\ M\\ S\\ R\\ B
\end{array}
\begin{array}{c}
V \quad C \quad F \quad A \quad E\ \quad D \quad W \quad M \quad S \quad R\ \quad B \\
\left[\begin{array}{c@{}c@{}c@{}c@{}c@{}c@{}c@{}c@{}c@{}c@{}c}
1 &\quad 1 &\quad 1 &\quad 1 &\quad 0 &\quad 0 &\quad 0 &\quad 0 &\quad 0 &\quad 0 &\quad 0 \\
1 &\quad 1 &\quad 1 &\quad 0 &\quad 0 &\quad 0 &\quad 0 &\quad 0 &\quad 0 &\quad 0 &\quad 0 \\
1 &\quad 1 &\quad 1 &\quad 1 &\quad 1 &\quad 0 &\quad 0 &\quad 0 &\quad 0 &\quad 0 &\quad 0 \\
1 &\quad 0 &\quad 1 &\quad 1 &\quad 1 &\quad 0 &\quad 0 &\quad 0 &\quad 0 &\quad 0 &\quad 0 \\
0 &\quad 0 &\quad 1 &\quad 1 &\quad 1 &\quad 1 &\quad 1 &\quad 1 &\quad 1 &\quad 1 &\quad 1 \\
0 &\quad 0 &\quad 0 &\quad 0 &\quad 1 &\quad 1 &\quad 1 &\quad 1 &\quad 1 &\quad 1 &\quad 1 \\
0 &\quad 0 &\quad 0 &\quad 0 &\quad 1 &\quad 1 &\quad 1 &\quad 1 &\quad 1 &\quad 1 &\quad 1 \\
0 &\quad 0 &\quad 0 &\quad 0 &\quad 1 &\quad 1 &\quad 1 &\quad 1 &\quad 1 &\quad 1 &\quad 1 \\
0 &\quad 0 &\quad 0 &\quad 0 &\quad 1 &\quad 1 &\quad 1 &\quad 1 &\quad 1 &\quad 1 &\quad 1 \\
0 &\quad 0 &\quad 0 &\quad 0 &\quad 1 &\quad 1 &\quad 1 &\quad 1 &\quad 1 &\quad 1 &\quad 1 \\
0 &\quad 0 &\quad 0 &\quad 0 &\quad 1 &\quad 1 &\quad 1 &\quad 1 &\quad 1 &\quad 1 &\quad 1
\end{array}\right]
\end{array}\!.
\end{eqnarray*}

Some of our hypotheses involved recent emergence or assembly of land masses. We used time-stratified models to represent these hypotheses, with the “areas available” matrix and their associated time periods to indicate which areas were available for occupancy during each time stratum. A single dispersal multiplier matrix was used across all time strata.

We fit models of historical biogeography using DEC (Ree et al. 2005; Ree and Smith 2008) models as implemented in the R package BioGeoBEARS (Matzke 2014), allowing a maximum of two areas per taxon. All DEC models have two degrees of freedom for the dispersal and extinction rate parameters. Model comparison was used to evaluate alternative hypotheses of historical biogeography, assessing model fits with the Akaike Information Criterion (AIC; Burnham and Anderson 2002). We assessed sensitivity of model selection to phylogenetic uncertainty (both topology and branch lengths) by fitting all of the range evolution models to each of the final 100 phylogenies of our Bayesian (BEAST2) phylogenetic reconstruction (Hill et al. 2022).

Using our best-fitting range evolution models, we estimated ancestral ranges and compared our results with the timings of terrane-accretion and island formation events (Davies et al. 1996, 1997; Pigram and Symond 1991). Reconstructed ancestral ranges were assigned to nodes by majority rule, and used to tabulate inferred range shifts, assigning the shift to the ancestral node along which it occurred. Although all asterophryine species are single-area endemics, we allowed two areas per taxon, as required by the software to return a model fit. We wrote a custom script to apportion the two-area probabilities equally between the original geogregions. For example, if the ancestral reconstruction assigned a 10% probability to a dual geogregion range comprised the EPCT + Fold Belt, half of the probability would be assigned to the EPCT and half to the Fold Belt. We plotted phylogenies annotated with model results using the R package ggtree (Yu et al. 2017).

We note that many biogeographic studies also fit DEC+J models (Matzke 2014), which allow an additional parameter for “jump” or long-distance dispersal. We found that DEC+J model fits on our data were degenerate as they returned models with zero dispersal and extinction probabilities, apportioning all range evolution to long-distance dispersal. Other authors have reported this model behavior, warning about spurious interpretations especially in situations where all lineages are restricted to single areas, as in our system (Matzke 2014; Ree and Sanmartín 2018). We, therefore, did not use DEC+J models in our study, as these results indicate a pathology of the model fit rather than a reasonable estimate of reality, which would be nonsensical given the poor dispersal abilities of frogs.

### Hypotheses

#### Island distance

The islands of the Bismarck, D’Entrecasteaux, and Louisiade groups lying off the northeast coast of New Guinea are surrounded by waters over 100 m deep and vary in distance to the mainland, which in turn should be inversely proportional to the probability of overwater dispersal. Therefore, absent any influence of geological history, nearby islands, such as New Britain and the D’Entrecasteaux Islands (<100 km away from the mainland), are expected to experience greater rates of dispersal than distant islands such as the Louisiade Archipelago (>200 km). We tested this idea by weighting the dispersal multiplier matrix by distance categories. Thus, the nearby D’entrecasteaux Islands (Normanby and Fergusson) and New Britain in relation to any mainland region are assigned a dispersal multiplier of 0.1, whereas Woodlark Island and the islands of the Louisiade Archipelago are assigned a dispersal multiplier of 0.001. Within-archipelago movement is assumed to be unconstrained (i.e., within the D’Entrecasteaux Islands, within the Louisiades + Woodlark), with a dispersal multiplier of 1.

#### Plate tectonics

Over the past 20M years, there have been significant land movements whose history may leave signatures in the present-day distribution of frog lineages. While it is well established that there are five major tectonic units that joined to form the New Guinea mainland, there are several geological hypotheses regarding the timing and order of their spatial connections. We constructed 11 alternative hypotheses (see Fig. 3 for explanation) to test these ideas. Our null or baseline hypothesis “Current Connectivity”(a) represents opportunities for dispersal based on present-day land connectivity, with no consideration of geological history. Here, mainland frogs can disperse freely across the main island of New Guinea, but overwater dispersal is unlikely. The next three alternative hypotheses represent the major competing ideas for the formation of the mainland: “Slow and Steady”, “Mobile Belt”, and “Recent Emergence” (see introduction for background; and Fig. 3b–d for descriptions of each scenario).

The remaining hypotheses explore scenarios for the history of the offshore islands and their relationship to the mainland. The origins of the Louisiade Archipelago have not been well studied, but based on petrological similarities, they are relatively old, being composed of forearc metamorphic rocks of at least Miocene age whose protoliths date back to the Cretaceous, and may once have been connected to other land masses, since their metasedimentary rocks are correlative to those in the current Owen Stanley Range of the Papuan Peninsula (Fig. 3e; Davies and Smith 1971; Pigram and Davies 1987; Baldwin et al. 2012). Prior to the opening of the Woodlark Basin, Woodlark Island was in close proximity to the Louisiade Archipelago (Fig. 3; Pigram and Davies 1987), possibly representing an element of the volcanic back-arc behind the Louisiade forearc (Webb et al. 2014). In its present-day position, Woodlark has been significantly displaced northward from its paleo-position, due to the rapid opening of the Woodlark Basin around 5 Ma. Another correlated hypothesis regarding the mountains of the Louisiade Archipelago is that they may represent the southern most extent of the ancestral Owen Stanley Range, which might once have been contiguous within the EPCT before its southeastern extension was isolated due to erosion and subsidence (Fig. 3g,h). This idea is again supported by petrological similarities (Davies and Smith 1971; Pigram and Davies 1987). We also include the D’Entrecasteaux Islands because of their very close proximity to the EPCT (although three islands comprise the D’Entrecasteaux group—Normanby, Fergusson, and Goodenough—we have frog genetic data from only the first two, so our subsequent discussions treat this unit as a two-island complex). The final hypotheses test whether the offshore islands share a history with either the EPCT or the Accreted Terranes, or both, as the latter lie on an active margin in proximity to the offshore islands (Fig. 3i–k).

#### Unconstrained DEC

As a point of comparison to the hypotheses informed by biogeographical evolutionary theory above, we also tested an “Unconstrained DEC” model with no dispersal multiplier nor time stratification. This model assumes that it is equally likely to transition between any two ranges.

All data and code necessary to reproduce all analyses and figures are provided in the supplemental online repository (Butler and Hill 2023) https://github.com/mbutler808/Hill_et_al_2023_IOB_Biogeography_Analysis.

## Results

The best-fitting overall hypothesis was “j: Offshore with Nearest Mainland”, and was superior to all other models. This result was robust to phylogenetic uncertainty, selected as the best model across the top 100 phylogenetic hypotheses (100% model selection frequency). The best model incorporates the “Slow and Steady” hypothesis for mainland assembly and a shared history between the offshore islands and their nearest mainland units (the Accreted Terranes for New Britain, and all others connected to the EPCT;  Table 2). Below we report the strength of evidence for the component ideas.

**Table 2:** Performance of alternative hypotheses for geographic range evolution. Model selection frequency for hypothesis *j: Offshore w/ Nearest Mainland* was 100% across 100 alternative phylogenies

Hypothesis	Subset	$-2log\mathcal {L}$	ΔAIC	D	E
j: Offshore w/ Nearest Mainland	b+island	996	0	0.0094	0.021
i: Offshore w/ EPCT	b+island	1002	6	0.0086	0.021
h: Historical Owen Stanleys	b+island	1006	10	0.0096	0.020
Unconstrained DEC		1019	23	0.0046	0.018
g: Owen Stanleys	b+island	1072	76	0.011	0.021
l: Island Distance	b+distance	1114	118	0.018	0.026
k: Offshore Islands w/ Accreted	b+island	1184	188	0.019	0.034
f: Woodlark w/ Louisiades	b+island	1285	289	0.027	0.025
e: Louisiade Archipelago	b+island	1334	338	0.028	0.023
b: Slow and Steady	mainland	1379	383	0.030	0.023
c: Mobile Belt	mainland	1398	402	0.011	0.026
a: Current Connectivity	mainland	1432	436	0.017	0.020
d: Recent Emergence	mainland	1686	691	0.025	0.058

We indicate whether hypotheses belong to the subset of mainland assembly hypotheses (mainland), or whether they build from the best-fit mainland model and explore island histories (b+island) or island distance (b+distance). For each hypothesis, the likelihood values (−2log$\mathcal {L}$) and difference in AIC from the best fitting model (ΔAIC) are given along with parameter estimates for the rate of dispersal (D) and extinction (E). Degrees of freedom for all models is 2.

### Mainland assembly

Comparing only the mainland assembly models, the distribution of Asterophryinae is best explained by the “b: Slow and Steady” hypothesis, which was far superior to the “c: Mobile Belt” and “a: Current Connectivity” hypotheses by 20 and 53 AIC units, respectively. “d: Recent Emergence” provided a substantially worse fit, by 309 AIC units. All of these mainland assembly models assumed the offshore islands remained detached.

### Island history

Building upon the “Slow and Steady” hypothesis for mainland assembly, we explored various hypotheses for the biogeographic history of the offshore islands. The second best overall model, by six AIC units, was hypothesis “i” in which all of the offshore islands including New Britain are connected to the EPCT (and not the Accreted Terranes; Table 2). Switching the shared history of all the offshore islands from with EPCT to with the Accreted Terranes provided a very poor fit, with a ΔAIC of 188 for hypothesis “k”.

The best model supports a shared history between Woodlark Island and the Louisiade Archipelago. We also tested several other models that compared Woodlark separate from the Louisaides versus joined together, and support was always improved by joining them. Model fit was further improved by connecting these to the EPCT, with the third best model (ΔAIC 10; Table 2) being “h: Historical Owen Stanleys,” which posits that the Louisiade Archipelago, Woodlark, and the D‘Entrecasteux Islands all originated as part of the Owen Stanleys. Therefore, with the exception of New Britain, we see a strong signal for even distant offshore islands sharing a history with the EPCT (Fig: 2, Table 1).

### Ahistorical and hybrid distance models

We tested three ahistorical models of interest. Hypothesis “a: Current connectivity”, implying that the current geography explains the distribution of Asterophryinae, was one of the worst fits to the data (ΔAIC 436; Table 2). In fact, the unconstrained DEC model (although not a top model with ΔAIC 23) was nevertheless a substantial improvement over current connectivity. A hybrid model which built on “b: Slow and Steady” for mainland assembly, but accounted for overwater distance to the offshore islands provided a poor explanation of the data with a ΔAIC of 118.

### Reconstructing the spread of Asterophryinae

**Fig. 4:**
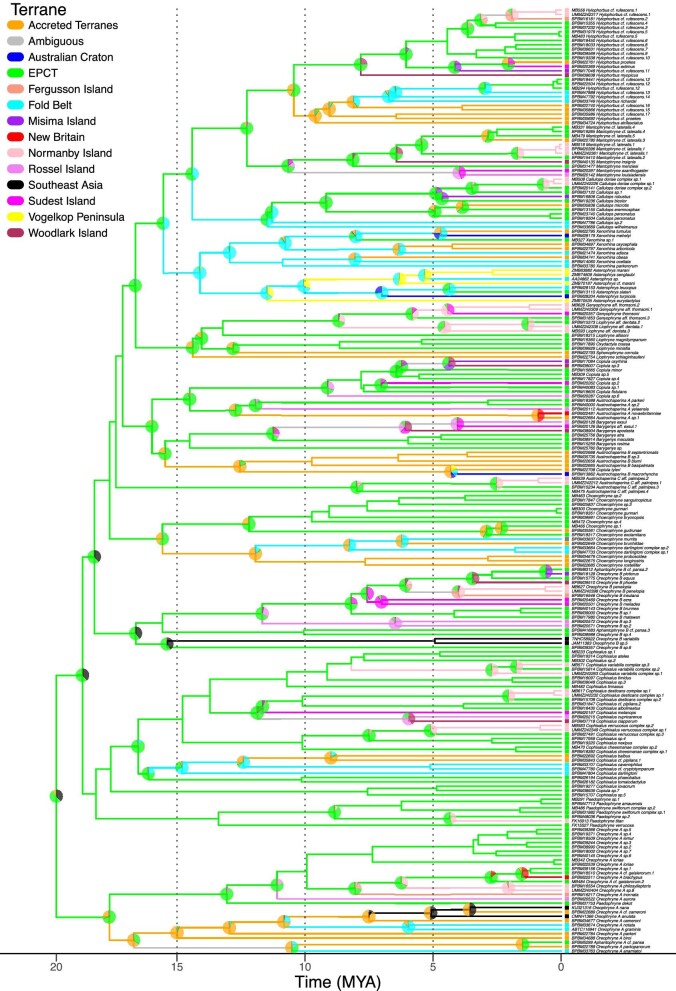
Reconstruction of Asterophryinae dispersal across New Guinea under the best fit DEC model. As all species are single-georegion endemics, any dual-area probabilities output by BioGeoBEARS are apportioned equally to single georegions.

From the best-fit model, we recover the EPCT as the center of origin of Asterophryinae placed at about 20 Ma (Feng et al. 2017; Hill et al. 2022), with subsequent spread throughout mainland New Guinea and across satellite islands, some more than 200km away (Fig. 4). We count a minimum of 71 georegion transitions required to explain the current distribution (42 between the mainland and islands, the remainder between mainland regions; Table 3). We discuss the implications of our findings below.

**Table 3:** Numbers of Asterophryinae dispersals through time between mainland and offshore islands of New Guinea

		*To*:										
		Mainland PNG					Islands				Mainland	Mainland
Time (Ma)	*From*:	E	A	F	V	C	L	W	D	B	Mainland	Island
20–15	E	–	3	3							6	0
15–10	E	–	4	1	–		9	2				
	A		–	3								
	F		1	–	1						10	11
10–3	E	–	1				8	4	11			
	A		–	–	1	1						
	F	2	3	–		1						
	V	–		–	–	1					11	23
3-present	E	–	2				1		5	1		
	A	–	–	–		–				1		
	F	–	–	–								
	V				–						2	8
Total											29	42

Mainland Terranes: E = EPCT, A = Accreted Terranes, F = Fold Belt, V = Vogelkop Peninsula, C = Australian Craton. Islands: L = Louisiade Archipelago, W = Woodlark Island, D = D’Entrecasteaux Islands, B = New Britain Island.

## Discussion

### The EPCT is the center of origin and ongoing diversification

The EPCT is a known center of endemism and species richness across many taxa. Although high mountains are present in both the Fold Belt and the EPCT, based on cicada relationships, Duffels (1983) concluded that the Papuan Peninsula (also known as the EPCT) was a separate biogeographic area, and not merely an eastward extension of the New Guinea central mountains. Furthermore, Boer and Duffels (1996) identified it as a discrete area of endemism within the Melanesian region. Kalkman et al. (2018) showed that the EPCT harbored a large number of endemic damselflies and concluded that a range expansion out of the EPCT gave rise to the rest of a species-rich clade of damselflies now found in New Guinea. This is plausible if they arrived from the EPCT to find much ecological opportunity on a still relatively depauperate and more recently uplifted New Guinea. Similarly, Polhemus and Polhemus (2004) noted that the EPCT supports an unusually rich biota of veliid water bugs representing numerous endemic genera and species not found in the remainder of New Guinea as a whole, while at the same time lacking certain endemic genera occurring in the main body of the island, indicating separate histories of faunal evolution followed by fusion of the two land masses. Kraus (2021) proposed that the opening of the Woodlark Rift and its ongoing extension has created a series of vicariance events promoting speciation in multiple lineages.

The EPCT itself is a composite terrane, which formed through multiple tectonic accretion events that began prior to its attachment to the remainder of New Guinea. This accretional history is significant in regard to its biotic evolution, as it may have brought communities together via collision, or at least within proximity so that dispersal to islands is possible. Although questions remain regarding the exact sequence of assembly within the EPCT, the concept of a composite land mass in eastern New Guinea formed by the collision of an Australian continental fragment and an island arc was advanced over 40 years ago in the geologic literature (Pieters 1978; Hamilton 1979), and has been subsequently supported by more recent research (Webb et al. 2014). Specifically, the basement rocks of southeastern New Guinea (the nascent EPCT) represent a displaced fragment of the Australian continental margin rifted away during the Cretaceous and moved northeastward by subsequent Paleocene-Eocene seafloor spreading (Zirakparvar et al. 2013). This fragment subsequently collided with a Late Paleocene-Early Eocene island arc to form the initial core of what would become the Papuan Peninsula (Davies and Warren 1988), leading to the emplacement of a broad ophiolite belt banked against the northern margin of this accreted unit. Such a scenario would imply that emergent land masses linked to island arcs may have been present in the vicinity of the current EPCT as early as the Paleocene, and that orogeny in the EPCT preceded that in central New Guinea by at least 10 My (Quarles van Ufford and Cloos 2005), making it one of the earliest high emergent land masses in the region. Thus, the fact that the EPCT is the center of diversity for many older lineages is understandable in the context of this early geological history.

**Fig. 5:**
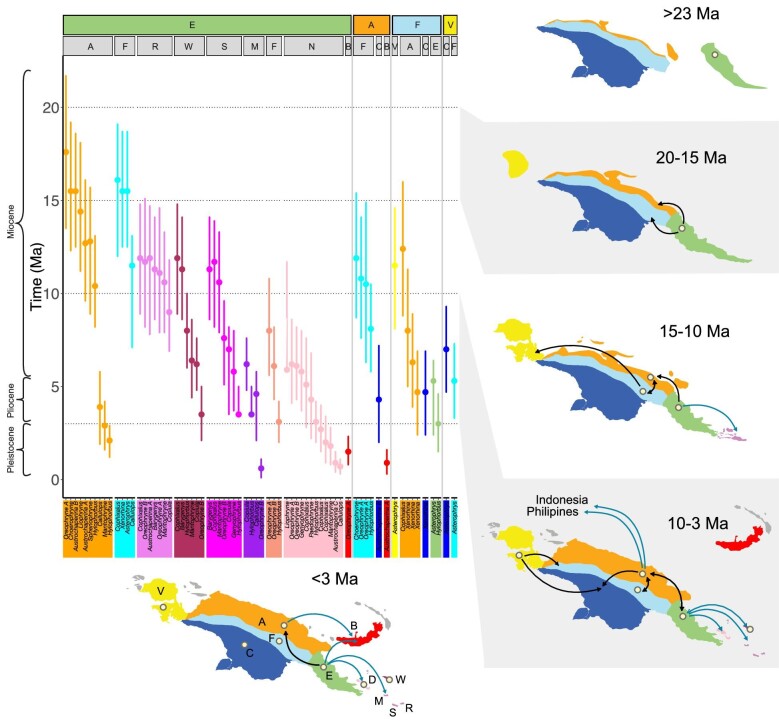
The dispersal of asterophryine frogs across New Guinea between terranes through time inferred from our phylogenetic analysis and the corresponding geological evolution. (A) Timings of independent dispersal events between terranes or islands inferred from DEC analysis (Fig 4). Genera involved are along the lower *X*-axis. The source terrane is indicated along the first row of the upper *X*-axis, arrival terrane or island along the second row. Points and genera blocks are colored by the arrival terrane/island. (E=EPCT in green, A=Accreted Terranes in orange, F=Fold Belt in light blue, V=Vogelkop Peninsula in yellow, R=Rossel Island in lilac, W=Woodlark Island in maroon, S=Sudest Island in fuscia, M=Misima Island in eggplant, G=Ferguson Island in coral, Y=Normanby Island in rose, and B=New Britain Island in red). (B)–(F) Corresponding land movements and the evolving islands. Beige dots indicate terranes/islands with frogs established at the start of the epoch. (B) Prior to 23 Ma, the EPCT is approaching the nascent New Guinea Island bringing the ancestors of Asterophryinae. (C) 23–20 Ma, the EPCT has docked onto the growing mainland, facilitating overland dispersal to the Accreted Terranes and the Fold Belt. (D) 15–10 Ma, the Vogelkop Peninsula docks, allowing dispersal. Dispersal continues between mainland terranes, on the southern tip of the EPCT the first genera disperse to Rossel, Woodlark, and Sudest Islands as they separate from the Owen Stanley Range, encouraging speciation. (E) 10–3 Ma, the Accreted Terranes have grown along the north coast, ongoing dispersal and speciation across land bridges and short water gaps until islands move further away. (F) The D’Entrecasteaux Islands (Normanby, Ferguson included in this study) have emerged very close to the mainland, and New Britain is on a collision course toward New Guinea, with the first dispersals to these islands.

**Fig. 6:**
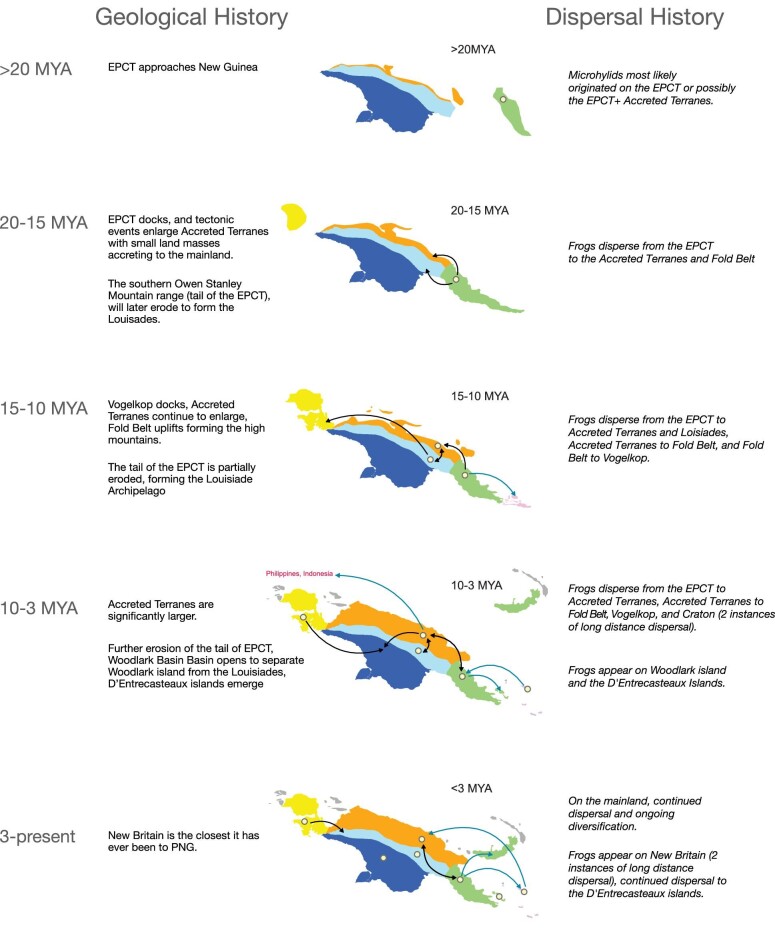
Narrative of the geological history of the mainland over the last  20MY (Davies 2012) and its offshore islands (Pigram and Davies 1987), along with dispersal pattern results from this study of Asterophryinae through time.

Our studies support this geological scenario, indicating that the EPCT is not only the origin of diversity for Asterophryinae, but a source for ongoing diversification and outward expansion (summarized in Fig. 6). Asterophryinae originated on the EPCT at least 20 Ma, as did 15 of the 18 genera (this study, Hill et al. 2022; Rivera et al. 2017; Feng et al. 2017), and entered the Fold Belt or the Accreted Terranes beginning around 17 Ma (Figs. 4, 5). This strong signal for an origin on the EPCT before later dispersing to the Accreted Terranes refutes the “Mobile Belt” hypothesis, which would predict a joint origin from both terranes. Instead, our results lend strong support to the “Slow and Steady” geological model of Pigram and Davies (1987); Davies (2012), a key prediction of which is that the Papuan mainland, except for the northern coast of the Accreted Terranes, was subaerial prior to the Early Miocene, with the EPCT docking onto the growing New Guinea composite land mass during the Late Oligocene to Middle Miocene (Davies et al. 1997). The dominance of the clade at low-mid elevation in the Early Miocene, as well as the exceptionally poor fit of the “Recent Emergence” model rejects the hypothesis of Hall (2002) that the bulk of the New Guinea mainland was submerged until 5 Ma.

What is more remarkable, however, is the coincidence of the timings of geological events and range transitions, and furthermore, the sheer volume of phylogenetically independent dispersals (71) across 18 genera. First, the docking of the EPCT to the Papuan mainland would have created new overland dispersal routes between major landmasses from the EPCT to the Accreted Terranes and the Fold Belt, bringing the first frogs to the growing New Guinea Island. We see that the vast majority of dispersals come out of the EPCT. Of the mainland to mainland dispersals, 48% originate from the EPCT, and dispersals to islands are nearly exclusively from the EPCT at 98%. Indeed in our biogeographic results, we see a wave of dispersal across the older genera of Asterophryinae in an “out of the EPCT to the north” pattern in: *Oreophryne A, Cophixalus, Choerophryne*, and *Austrochaperina B* in relation to *Baryengys* (Fig. 5), with each genus independently splitting into EPCT versus Accreted Terrane/Fold Belt divisions at the same point in asterophryine history (Fig. 4), leading to major subclades with ongoing diversification. In the discussion that follows, we acknowledge that both the phylogenetic dating and geological dating are approximate. Nevertheless, we recover very similar date ranges along independent branches of the phylogenetic tree, and these range shifts coincide with the approximate dating of geological events are remarkable.

### The coincidence of geology with dispersal

About 12 Ma, there are multiple major geological events: orogeny of the central cordillera, docking of the Vogelkop Peninsula, and the opening of the Woodlark Basin. The orogeny of the central mountains of the Fold Belt began about 12 Ma (Pigram and Davies 1987; Pigram and Symond 1991; Davies et al. 1996; Quarles van Ufford and Cloos 2005; Baldwin et al. 2012; Davies 2012). Transitions from the EPCT into the Accreted Terranes and/or the Fold Belt tapered off ∼12 Ma (Fig. 5), when the central mountains may have risen high enough to become a geographic barrier for some lineages. Members of the genera *Choerophryne, Oreophryne A*, and *Hylophorbus* moved from the Accreted Terranes to the Fold Belt ∼12–8 Ma, as the mountains were rising. A final burst of transitions of three genera from the EPCT to the Accreted Terranes occurred ∼4 Ma, with the docking of the Huon–Finisterre–Adelbert blocks of the Bismarck Plate, which must have arrived without any preexisting Asterophryinae species. Contemporaneously in time, ∼14 Ma, the genus *Asterophrys* expanded from the Fold Belt to the Vogelkop Peninsula, coincident with the docking of the Vogelkop Peninsula (Pigram and Davies 1987; Polhemus and Polhemus 1998; Quarles van Ufford and Cloos 2005; Davies 2012), rather than the more recent date of 7–3 Ma proposed by Holm et al. (2019). The timing of these transitions closer to the mid-Miocene further supports the “Slow and Steady” model rather than the “Recent Emergence” model (Hall 1998, 2002).

**Fig. 7:**
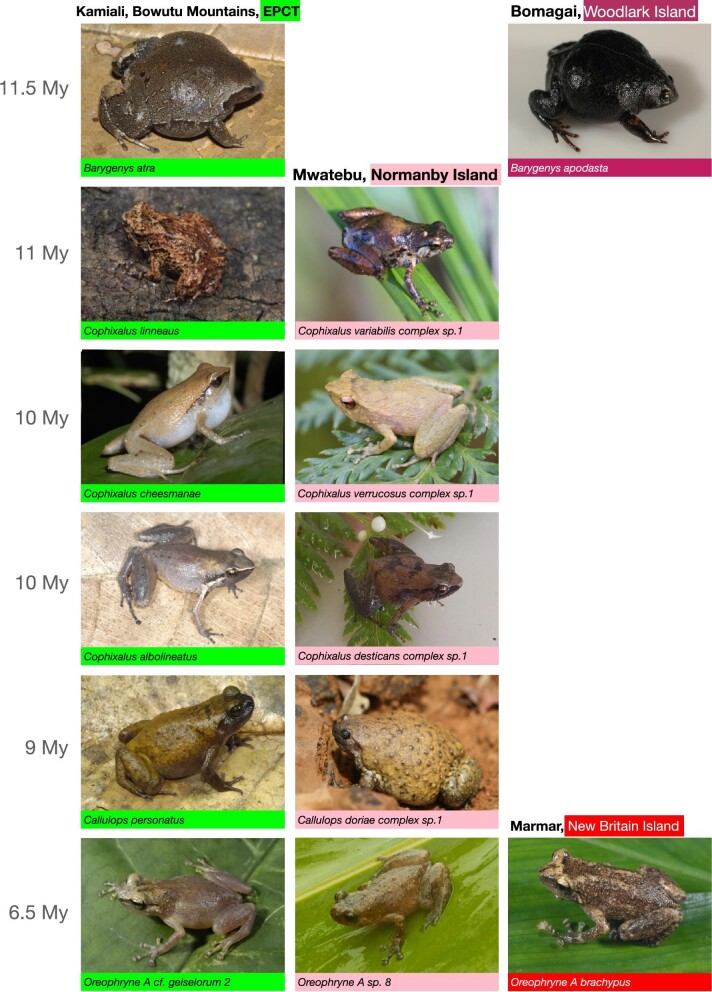

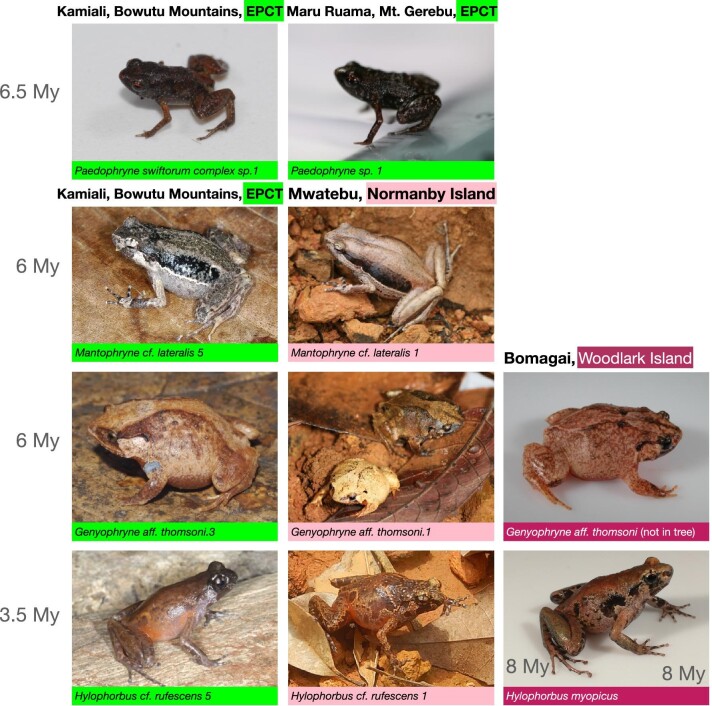
Examples of asterophryine frog species groups, which illustrate the range transition across the EPCT to the offshore islands with subsequent speciation. Species names are provided below each plate, with the site across the tops of the columns of plates, and time to the most recent common ancestor on the left of each row of plates. *Hylophorbus myopicus* of Woodlark Island, however, is separated by 8 My from *Hylophorbus* of Kamiali, EPCT and Normanby Island. Photo credits: Normanby Island and Maru Ruama, EPCT frogs were provided by M. Butler, whereas Kamiali, EPCT, Woodlark Island, and New Britain Island photos were provided by A. Allison. Please note that these are photos from sites visited by the authors and not necessarily the closest sister taxon pairs.

Along the southern tip of New Guinea, also beginning about ∼12 Ma, was the rifting off of the southern end of the Owen Stanley Range in the EPCT into the Louisiade Archipelago and Woodlark Island. We see a very strong signature of this event in our model fits, as any model linking the Louisiade Archipelago and Woodlark to the EPCT provides a far superior fit to similar models without this motif. At this time, the EPCT would have presumably been fully populated with potential source populations for dispersal across small water gaps or may have already populated the area that became newly isolated islands. This scenario would readily explain the skewed distribution of 12 range transitions involving 7 genera coincident in time to these islands, concentrated around the 12 Ma mark, followed by continuing range transitions that taper off as the islands would be rifting farther away. It would also explain why the species on these islands were independently seeded from the EPCT, and not arising by rare dispersal followed by in-situ diversification, and also explain why species do not disperse in a stepping stone manner to the islands. Indeed, it is remarkable that all species have closest sisters on the EPCT, and in-situ radiation on the islands is rare (plates of some of pairs of relatives across the EPCT and offshore islands are shown in Fig. 7a,b).

Finally, the recent rise of the D’Entrecasteaux Islands and the close approach of New Britain Island demonstrate that at very close distances, oceanic dispersal is possible even for terrestrial frogs, as lineages on these nearby islands, which were never connected are related to sisters on the EPCT.

The best-fitting overall hypothesis was “j:Offshore Islands with Nearest Mainland”, which is a refinement of “Slow and Steady” and supports these geological scenarios regarding the origins of the offshore islands discussed below. Thus, the hypderdiversity of this low-dispersal clade can be explained by major land movements that provide multiple vicariance events through time, which allowed numerous genera to simultaneously speciate (Fig. 5), and lend strong support for the role of geology in driving ongoing diversification.

#### Long-distance dispersal versus carriage by plate tectonics

With the large number of inferred range transition events (71 transitions), a naive interpretation would be that dispersal is frequent. However, phylogenetic evidence confirms that these species are poor dispersers. In addition to the numerous instances of cryptic species within Asterophryinae (i.e., phylogenetically distinct sister lineages in close spatial proximity (Hill et al. 2022)), from our DEC modeling, we recover an overall rate of extinction that is higher than the rate of dispersal, consistent with a clade of low-dispersing organisms that tend to readily fragment by isolation. Furthermore, with regard to the mainland tectonic units, if long-distance dispersal were common, an unrestricted model allowing free dispersal between any two mainland units should be favored. Instead, our results show that a model limiting dispersal to adjacent tectonic units was far superior (“Slow and Steady” fit better than “Current Connectivity” by 53 AIC units). Nearly all range shifts occur in a single step (Fig. 5). Where a lineage passes through multiple terranes, they do so in a stepwise manner: the ancestor of *Asterophrys* and *Xenorhina* originated on the EPCT, then dispersed to the Fold Belt, then to the Vogelkop peninsula. All of these findings point to the difficulty of long-distance dispersal. However, the frequency of range transitions can be understood in the context of plate tectonics.

Especially with regard to the offshore islands, we originally thought that our phylogeny (presented in: Hill et al. 2022) must be wrong. How could small terrestrial frogs disperse over hundreds of kilometers of open ocean, separated by 10 MY from their nearest sister lineage on the EPCT? Frogs are notoriously poor oceanic dispersers (Duellman and Trueb 1994), which is supported by experimental evidence with adult anurans suffering toxicity at $80\%$ of ocean salinity, and embryos developmentally impaired if exposed to only $25\%$ of ocean salinity (Gordon et al. 1961; Seymour 1994; Hopkins et al. 2012). Yet situations are known where rare oceanic dispersal must have occurred, such as on the Comoro Archipelago situated between Madagascar and Africa, which is home to two endemic anuran species but with its volcanic origin has neither been connected to Madagascar nor Africa (Vences et al. 2003), and in this study, with regard to the D‘Entrecasteaux Islands and New Britain.

In the present study, we did not find that frogs dispersed in a stepping-stone fashion from nearer to more distant islands (the Island Distance model was a very poor fit with ΔAIC 118; Table 2), as would be predicted by Island Biogeography Theory (MacArthur and Wilson 1967; Losos and Ricklefs 2009). All source populations are directly from the mainland. Furthermore, we see no evidence for community-wide vicariance. Communities on islands are generally composed of individual representatives of multiple genera, and therefore, species within a community are connected by deep histories (Hill et al. 2022), and furthermore, each species is sister to taxa from different source populations on the mainland. If there were evidence of community-wide vicariance, we would expect that models that group archipelagos together such as “e: The Louisiade Archipelago” would provide a much better fit.

On the other hand, if islands were always separated requiring rare over-water dispersal, we would expect the “a: Current Connectivity” hypothesis to provide a better fit. Instead, it provides amongst the worst fit. Intriguingly, it is a substantially worse fit than the unconstrained DEC model, which makes no assumptions about connectivity and, therefore, implies that all regions are equally connected. The answer that emerges from the amalgamation of evidence is that the frogs in most cases dispersed over land connections or very narrow water gaps, which themselves evolved over time. One possibility is that in rare events, frogs may have rafted across on fallen trees, which can happen during monsoons or tropical storms. Nevertheless, dispersal over narrow water gaps would allow arrivals from independent source populations for as long as the island was within close proximity, which may change with plate tectonic movements.

#### The Louisiade Archipelago

These islands are an excellent case in point for diversification driven by vicariance via plate tectonic movements, being distant offshore islands that are home to over 18 candidate species of Asterophryinae (Boulenger 1890; Méhelÿ 1901; Zweifel 1963, 1972; Zweifel and Tyler 1982; Burton 1986; Zweifel 2000; Richards and Oliver 2007; Kraus and Allison 2009; Kraus 2016), yet separated by over 200 km of saltwater barrier from any potential source population. Rather than inferring 18 independent dispersals within the last 12 million years, an alternative hypothesis is that they may have fragmented from the Papuan mainland. If dispersal was to a remote oceanic island at fixed distance from the source population, we would expect to see a random pattern in the timing of dispersal events. Instead, the data show that the dispersals, all originating from the EPCT, are highly concentrated at ∼12 Ma, followed by a tapering off until ∼4 Ma, consistent with islands fragmenting off of a mainland source (Fig. 5). Geological evidence supports this scenario. Petrological samples taken from the Louisiade Archipelago are similar in composition to those of the Owen Stanley Range on the EPCT indicating a shared geological history between these two seemingly distinct landmasses (Pigram and Davies 1987; Baldwin et al. 2012; Davies 2012).

In the work of Pigram and Davies (1987), the Louisiade Islands are shown as a disjunct sector of the Owen Stanley Terrane, most of which lies in the interior of the Papuan Peninsula. In their current form, they represent the partially drowned remnants of an old mountain range that ran down the middle of what is now the EPCT, and the metasedimentary rocks of the Louisiade Archipelago have been correlated with those of the Owen Stanley Range (Davies and Smith 1971). The protoliths of these metasedimentary rocks are Cretaceous volcaniclastic sediments derived from the eastern Australian continental margin (Zirakparvar et al. 2013), and the metamorphic rocks of the Lousiades have been interpreted to represent mid-Cretaceous sediments scraped off of a subducting plate and incorporated into an accretionary wedge in the fore arc of a southward-migrating Miocene volcanic arc over northward dipping subduction. Woodlark Island may represent a remnant of the volcanic back-arc associated with this system (Webb et al. 2014). These scenarios would imply that emergent land masses may have been present in the Louisiades sector since at least the Early Miocene.

#### Woodlark Island

This island is currently >200 km from the Papuan mainland, lying in an isolated position to the northeast, and also contains a surprising number of Asterophryinae species, all with sister taxa on the EPCT (Fig. 4). Pigram and Davies (1987) treated Woodlark as lying on a separate terrane from the Louisiades, but noted that it had a basement of Eocene age, overlain by Oligocene limestones. Webb et al. (2014) suggested that Woodlark Island might represent a remnant of the volcanic back-arc associated with the Miocene arc that formed the Louisiades, which is consistent with its basement of pre-Miocene basalts reported by Ashley and Flood (1981), indicating that Woodlark and the Louisiades may have had a linked tectonic history. One hypothesis is that Woodlark was adjacent to the Louisiade Archipelago (Pigram and Davies 1987; Baldwin et al. 2012), prior to the opening up of the Woodlark Basin, which formed by rifting that initiated in the Late Miocene and continues to the present time (Webb et al. 2014). This rifting displaced the submarine ridge that Woodlark sits on to the northeast, pushing it away from its previous proximity to the Louisiades. This geological model is consistent with the large number of independent frog dispersals to Rossel (currently the most easterly of the Louisiades), Sudest, and Woodlark Islands, between ∼12 and 8 Ma, consistent with the hypothesized timing of fragmentation among these three islands. Thus, the initial proximity of Woodlark Island to the Louisiades provided opportunities for dispersal and differentiation, which tapered off as the oceanic gap widened. Misima island, which is the most westerly of the Louisiades and nearest to New Guinea, has a later history of dispersal from the EPCT, starting around ∼6 Ma, consistent with a more recent separation.

#### Southeast Asia

The historical genus *Oreophryne* was recently identified as two distinct and unrelated genera *Oreophryne A*, which is the oldest genus of Asterophryinae, and a younger, unrelated clade *Oreophryne B* (Hill et al. 2022). We have four *Oreophryne* samples from this region, two species in *Oreophryne A* from the Philippines, which are sister to species on the Accreted Terranes, and two species in *Oreophryne B* from Sulawesi, which are sister to species on the EPCT. McGuire et al. (2023) has proposed overwater dispersal to Sulawesi in flying lizards. While we cannot rule out overwater dispersal for frogs, dispersal to Sulawesi could be facilitated by westward-motion along the Pacific plate boundary, where accommodation of convergence has sheared fragments from northern New Guinea and sent them westward toward the Sulawesi region (Hamilton 1979; Polhemus 2007) along a series of left-lateral fault zones. These cases suggest an out-of-PNG to the Sunda Arc dispersal route hypothesis. Recently, several additional genera of Asterophryinae from Southeast Asia have been described: *Siamophryne, Gastrophrynoides*, and *Vietnamophryne* and were found to be basal sisters of the Papuan Asterophryinae (Poyarkov et al. 2018; Suwannapoom et al. 2018). This was interpreted as evidence for a “down-from-Southeast-Asia” pattern of dispersal (Tyler 1979). More sampling of species as well as inclusion of nuclear genes in phylogenetic analyses is required to resolve the structure of the Southeast Asian clades in relation to the Papuan clades and to determine whether such faunal disjunction might be linked to much earlier tectonic events.

#### Limited over water dispersal

Several islands contain Asterophryinae species but have never been connected to the New Guinea mainland, and as such must have been colonized by over-water dispersal. These include the D’Entrecasteaux Islands and New Britain in the Bismarck Archipelago. Asterophryines are absent from the other islands in the Bismarck Archipelago, viz. New Ireland, New Hanover, and Manus islands.

#### The D’Entrecasteaux Islands

The D’Entrecasteaux group of islands lies close to the Papuan Peninsula of New Guinea (40 km from the EPCT; Table 1), and contains an exceptional diversity of 18 candidate species on Normanby Island, with more modest assemblages on Fergusson and fewer yet on Goodenough. We note that we lack genetic samples from Goodenough, but we know the environment is a bit more xeric and less suitable for frogs. Asterophryinae dispersal to the D’Entrecasteaux Islands began ∼8–5 Ma and continues to the present (Fig. 5). These islands are an emergent metamorphic core complex, consisting of gneiss and amphibolite domes with granodiorite cores (Pigram and Davies 1987), and with the potential exception of part of Normanby, were never connected to the mainland. They are not volcanic features, but instead seem to represent incipiently subducted continental crust that has rebounded back up through the zone of weakness at the west end of the Woodlark Rift (Baldwin et al. 2008; Wallace et al. 2014). They are also relatively new islands, of Pliocene age or later (Abers et al. 2002), and are still rising and enlarging. So unlike the Louisiades, which are old islands that are becoming progressively smaller, the D’Entrecasteaux group consists of young islands that are becoming progressively larger. As such, they are a very recent colonization target that has required overwater dispersal to reach.

Goodenough and Fergusson Islands are internally cohesive emergent metamorphic units, while Normanby Island has a more complicated and potentially composite history. The island is divided by a right-lateral strike-slip fault (Wallace et al. 2014). The narrower western section of Normanby, to the north of this fault, is of similar composition and origin to Goodenough and Fergusson Islands, while the larger, furrowed eastern section is a separate lithological unit that is more allied to parts of the Owen Stanley uplift in the central Papuan Peninsula (Baldwin et al. 2012). Thus, the section of Normanby where our samples were collected, with a surprising species richness (18 candidate species), may be of separate origin more closely linked to the EPCT, possibly representing a younger version of the situation in the Louisiades. The different tectonic histories of Normanby versus Fergusson and Goodenough help to explain the species diversity disparities, while providing evidence that overwater dispersal can occur when sufficiently short distances are involved.

#### New Britain Island

Currently there are only two species of Asterophryinae known to be present on New Britain, *Austrochaperina A novaebritanniae* whose sister taxon is from the Accreted Terranes, and *Oreophryne A brachypus* with a sister taxon on the EPCT. These are clearly two independent dispersal events within the past 3 Ma. Given that New Britain is the largest offshore island and also among the closest (Table 1), the question should be why isn‘t it home to more species? One hypothesis is that the genus *Cornufer* of the family Certatobatrachidae likely arrived first to New Britain and has radiated extensively (Brown et al. 2015), possibly excluding microhylids. Another possibility is dispersal limitation. Considering that New Britain is on a collision course with the Papuan mainland, and currently lies as close as it ever has to New Guinea (Kroenke et al. 1984; Pigram and Davies 1987; Polhemus and Polhemus 1998; Quarles van Ufford and Cloos 2005; Baldwin et al. 2012; Davies 2012). In this case, overwater dispersal is the only mechanism available to frogs, and the distance of ∼80km may represent an overwater dispersal limit. The low number of asterophryne species despite its large land area makes sense in this context, but this situation certainly merits further study.

### A comment about model-based approaches to biogeography

We employed a phylogenetic model-based approach to explore hypotheses about historical biogeography. That is, we constructed a series of models that approximate our hypothetical phenomena (i.e., distance, various geological scenarios), and allowed them to compete for the best explanation of the data. Luckily, despite the complexity of hypotheses involving partial areas, it appears that our model fits are additive such that for example—the addition of Woodlark Island to the Louisiade Archipelago produces the same improvement of fit regardless of the assumptions of other parts of the model. We caution that this need not be the case, and care must be taken in making such assessments. Nevertheless, the gains for biology in rigorous interrogation of models are tremendous. By comparing models head-to-head, we could clearly identify which ideas were supported by data and which were not.

It has been noted by Ree and Sanmartín (2018) that many studies do not exploit the power of model specification flexibility afforded by the DEC class models. Rather, most studies limit the comparison to major classes of models (i.e., DEC, DEC+J, and DIVA), but do not vary the model to approximate the actual phenomena of interest, leaving conclusions to interpretation of the ancestral range reconstructions. Interpretation of model results is, of course, very important, but we agree that the most commonly used strategy represents a missed opportunity to use the power of statistical model-comparison approaches to drill down into the exploration of the data. We can use a hypothesis-driven phylogenetic approach to differentiate between customized hypotheses to test specific biological or geological phenomena.

### Conclusion

We find using an explicit model-based approach reveals clear signals in the data with very strong support for each of these model additions: the pairwise connections of the mainland, and the affiliation of the Louisiades+Woodlark with the EPCT, as well as the D’Entrecasteux Islands. Together with the interpretation of the ancestral range reconstructions and their coincidence in timing with geological events, they support the “Slow and Steady” geological hypothesis for the formation of the region (Pigram and Davies 1987; Davies et al. 1996; Polhemus and Polhemus 1998; Quarles van Ufford and Cloos 2005; Davies 2012), with the EPCT as the center of origin and source for ongoing diversification. In a group with impossibly complex distribution yet with poor dispersal ability to islands with no current connection to mainland, we need not accept wild dispersal scenarios. We can rigorously test geological ideas to find a strongly supported biogeographical model involving dispersal over land connections (in multiple varieties of joining and fragmenting) or over narrow water gaps to explain the convoluted dispersal pathways and exuberant diversity of the Asterophryinae.

Importantly, we find important consequences for biodiversity in the fact that the earth’s surface is not fixed but itself evolves. Not only can land area change over time, sometimes increasing by volcanism or shrinking through erosion, but the distances between land masses can evolve as well, changing the degree of isolation through time and providing another axis upon which geological evolution can alter the dynamics of biotic evolution.

## Supplementary data

All data and code to reproduce this analysis are available from the Digital Repository: https://github.com/mbutler808/Hill_et_al_2023_IOB_Biogeography_Analysis with DOI: 10.5281/zenodo.7996619.
